# Clinical history-taking and physical examination in medical practice in Africa: still relevant?

**DOI:** 10.3325/cmj.2016.57.605

**Published:** 2016-12

**Authors:** Ayo Oyedokun, Davies Adeloye, Olanrewaju Balogun

**Affiliations:** 1 Saint Nicholas Hospital, Lagos, Nigeria *ayooyedokun@yahoo.com*; 2 Demography and Social Statistics, and the e-Health Research Cluster, Covenant University, Canaanland, Ota, Ogun State, Nigeria; 3 Centre for Global Health Research and the World Health Organization’s Collaborating Centre for Population Health Research and Training, The University of Edinburgh Medical School, Usher Institute, Edinburgh, UK; 4 Department of Surgery, Lagos University Teaching Hospital, Lagos, Nigeria

Advancement in technology is continually redefining the practice of medicine globally, with sophisticated diagnostic machines, investigative procedures, and medical tests now widely available for medical diagnosis. Indeed, new discoveries, devices, and the internet have positively influenced the way physicians diagnose, treat, and palliate diseases. However, unlike in many high-income settings, where advancements in medical technology have, in most cases, been complementary to understanding disease pathogenesis and acquisition of core clinical skills, emerging evidence from many low- and middle-income countries, especially Africa, point to the contrary (1,2). There are widespread concerns about doctors’ declining bedside skills and clinical aptitude (3). While it is a requisite that medical students and doctors-in-training must demonstrate competence in history-taking and physical examination, there are reports that doctors fail to maintain and improve on these skills over their professional practice owing to over-reliance on results of medical tests (3). In Africa and many resource-constrained settings, where majority can hardly afford simple medical tests, the essence of detailed history-taking and physical examination cannot be overemphasized. It is therefore important to consider if improvements in health technology and medical diagnosis, and their introduction in many African settings, should alter the role of the fundamental history-taking and physical examination in general clinical assessment.

## Clinical diagnosis in medical practice

A systematic approach to the bedside examination of a patient is essential to determine the significance of an abnormal physical finding. It aids critical thinking, actively searches for signs of disease, identifies possible diagnosis, and helps in making tentative management plans (1-4). Sackett and Rennie reported that a doctor’s first few minutes of history-taking and physical examination are packed with audio-visual and tactile information about the patient, which may subsequently determine the path of diagnosis and effectiveness of treatment given (5).

Clinical reasoning based on facts elicited from symptoms and signs in the history-taking and examination has to be tested against basic scientific background and knowledge acquired during medical training (6). Laboratory results may therefore be seen as supporting evidence and not dictate over a meticulous clinical evaluation (6). In fact, the reliability of any laboratory information depends on the state of the medical equipment and the technologist’s ability to use it effectively (7). In some African settings, doctors order several tests to make diagnosis without considering this fact and some other basic evidence from clinical research relevant to their environment (2). Results may be false positive or false negative, and in such cases, a lack of basic understanding of the disease presenting complaints may result in instituting a wrong treatment plan (7). In addition, this may result in incurring unnecessary costs by the patient and overstretching the limited health resources in these settings. Moreover, ordering tests without detailed history-taking and physical examination tends to strain patient-doctor’s relationship (7). In the absence of trust and confidence on the part of the patient, the effectiveness of most therapies fails. It is therefore important that physicians remain purposeful in the care of patients and uphold strict professional conduct. A detailed interaction and observation during consultations cannot be replaced by laboratory results.

## History-taking and physical examination versus investigations

It has been widely reported that history-taking and physical examination in medical practice may have been declining across Africa due to poor consultation venues, overcrowded clinics with few doctors to patients, poor salary packages for doctors, and the fact that to augment income, doctors often rely on bonuses and re-imbursements offered by manufacturers and pharmaceutical companies for using their diagnostic equipment and drugs (1,4). This practice has even led to a wrong perception among patients that laboratory tests and investigations must precede any diagnosis and/or treatment. It is now not uncommon for patients to walk into medical laboratories or radio-diagnostic centers to have investigations done even before consulting with a doctor. Invariably, the component of history-taking and physical examination as contained in the holistic management of patient – consideration of the complete physical, psychological, social, and spiritual factors in the management and prevention of disease – is gradually fading in the region (2,8). Studies have consistently shown that over-reliance on technology may not necessarily improve the quality of patient care (7). Hence, experts have argued that the justification for any investigation in medical practice ought to be complementary – answering specific questions related to history-taking and physical examination already done (1,4). For example, Reilly (4) reported that physical examination had substantial effects on the care of medical inpatients, with one-in-four patients ordered for investigation already having pivotal physical findings necessary for diagnosis. Some studies conducted in the United States, India, and Brazil also reported that history-taking was responsible for 76%, 78.6%, and 77.8% of all diagnoses made, respectively, and that investigations played complementary roles in excluding other diagnostic options and increased physicians’ self-confidence (6-8). In fact, Dooley et al (9) explained that detailed clinical history remained the mainstay of diagnosis for most of the critical cases of pediatric neurology they encountered in their practice. Additionally, a less equipped primary health care center may still arrive at a correct diagnosis in about 88% of cases following brief history-taking and physical examination, and treatment can be commenced based on these findings (5).

However, it is important to note that some patients do require thorough investigations to arrive at a definitive diagnosis and institute appropriate care (8). In a study among patients with developmental delay, Shevell et al (10) found medical investigations and imaging to be helpful in determining etiologic diagnosis in about 30% of cases. Khunti et al (11) also emphasized that an objective investigation including laboratory tests, electrocardiogram, and echocardiogram must be performed in patients with clinically suspected heart disease. Diacon (12) also noted that physician’s clinical acumen may not predict the accuracy of selected puncture sites, but that a bedside ultrasonography potentially increases the yield and reduces complications during diagnostic pleurocentesis. It is also worthwhile to note that some investigations, asides from being diagnostic, may also be therapeutic, as is the case in endoscopies performed for some gastrointestinal, respiratory, or urinary tract diseases. Our view however is that while some investigations may be necessary and very important in overall clinical management, this can still not replace a detailed medical history-taking and physical examination during consultations. Moreover, in many African settings, where patients can hardly afford costs of medical care, needless laboratory tests may be avoided to help reduce medical expenses. Positive management outcomes have been reported following scenarios where physicians used few investigations and tests owing to patient’s financial constraints and lack of insurance schemes (12).

## Conclusion

We understand that our report may be incomplete due to the absence of original information, time variations, and precise quantitative data and analysis of the application of health technology, history-taking, and physical examination across many African settings. Moreover, consideration of widespread differences in medical practice among various African contexts may have further improved our report, as we still cannot characterize with all certainty the difference in the use of the health technology between Africa and other world regions. The lack of original research appraising core clinical skills and health technology across many African countries is a major factor, and prospective studies are still constrained by lack of funds to conduct large population-representative studies from which evidence-based decisions may have emerged. As practicing physicians however, we support the opinion that technology may serve as an extension of a doctor’s core clinical duties and not be regarded as a replacement. We present this report to raise awareness on the relevance of history-taking and physical examination in the continent. We hope this will be re-emphasized in health policy and clinical curriculum guiding the training of medical students and resident doctors, and overall medical practice across many African countries.
